# Contrast Sensitivity Impairment in Diabetic Retinopathy: Clinical and Structural Correlates

**DOI:** 10.3390/diagnostics16152401

**Published:** 2026-07-30

**Authors:** Laura Andreea Ghenciu, Diana Andrei, Alexandra Magdalena Ioana, Roxana Stoicescu, Daniela Iacob, Emil Robert Stoicescu, Sorin Lucian Bolintineanu

**Affiliations:** 1 Department of Functional Sciences, Discipline of Pathophysiology, “Victor Babeș” University of Medicine and Pharmacy, 300041 Timisoara, Romania; bolintineanu.laura@umft.ro; 2 Center for Translational Research and Systems Medicine, “Victor Babeș” University of Medicine and Pharmacy, 300041 Timisoara, Romania; 3 Department of Balneology, Medical Rehabilitation and Rheumatology, “Victor Babes” University of Medicine and Pharmacy , 300041 Timisoara, Romania; 4 Doctoral School, “Victor Babes” University of Medicine and Pharmacy Timisoara, Eftimie Murgu Square No. 2, 300041 Timisoara, Romania; alexandra.ioana@umft.ro; 5 Department of Anatomy and Embryology, “Victor Babes” University of Medicine and Pharmacy Timisoara, 300041 Timisoara, Romania; roxana.iacob@umft.ro (R.S.); s.bolintineanu@umft.ro (S.L.B.); 6 Department of Neonatology, “Victor Babes” University of Medicine and Pharmacy Timisoara, Eftimie Murgu Square No. 2, 300041 Timisoara, Romania; iacob.daniela@umft.ro; 7 Radiology and Medical Imaging University Clinic, “Victor Babes” University of Medicine and Pharmacy Timisoara, Eftimie Murgu Square No. 2, 300041 Timisoara, Romania; stoicescu.emil@umft.ro; 8 Field of Applied Engineering Sciences, Specialization Statistical Methods and Techniques in Health and Clinical Research, Faculty of Mechanics, “Politehnica” University Timisoara, Mihai Viteazul Boulevard No. 1, 300222 Timisoara, Romania; 9 Research Center for Pharmaco-Toxicological Evaluations, “Victor Babes” University of Medicine and Pharmacy Timisoara, Eftimie Murgu Square No. 2, 300041 Timisoara, Romania

**Keywords:** diabetic retinopathy, contrast sensitivity, visual function, retinal neurodegeneration, optical coherence tomography, diabetic macular edema, OCT angiography, retinal biomarkers, visual acuity, diabetes mellitus

## Abstract

Diabetic retinopathy (DR) is one of the main causes of vision impairment globally, and it is increasingly recognized as a neurovascular disease characterized by both microvascular and neural dysfunction. Traditional assessment approaches primarily focus on structural retinal alterations and visual acuity, which may fail to identify early functional impairments. Contrast sensitivity (CS), which measures the capacity to distinguish subtle luminance differences, is increasingly recognized as an early manifestation of retinal dysfunction in diabetes mellitus. This narrative review seeks to describe current knowledge on the role of CS in DR, including its pathophysiological foundation, clinical importance, and link to structural retinal alterations. A systematic literature search of PubMed, Scopus, and Google Scholar (2000–2026) was performed to identify studies on CS in diabetes mellitus and diabetic retinopathy. Multiple investigations found that diabetic patients, including those with no clinically evident DR and normal visual acuity, had lower contrast sensitivity. CS impairment was associated with retinal neurodegeneration, ganglion cell dysfunction, microvascular alterations, and diabetic macular edema severity. OCT and OCT-A findings showed important structure–function correlations between reduced CS and retinal thinning, capillary dropout, and impaired perfusion. CS testing may provide additional functional information beyond standard visual acuity assessment and may improve early disease detection and monitoring. In conclusion, contrast sensitivity represents a promising functional biomarker for early diabetic retinal dysfunction and may complement structural imaging and visual acuity testing in DR evaluation. Although methodological variability currently limits widespread clinical implementation, advances in digital technologies and multimodal assessment strategies may facilitate its future integration into routine ophthalmologic practice.

## 1. Introduction

Diabetes mellitus represents one of the most significant global health challenges, with its prevalence continuing to rise at an alarming rate [1]. Among its microvascular complications, diabetic retinopathy (DR) remains a leading cause of visual impairment and blindness in the working-age population worldwide [2,3]. Traditionally, the clinical evaluation and staging of DR have relied heavily on structural retinal changes identified through fundus examination and imaging techniques [4,5,6]. However, these approaches may fail to capture early functional deficits that precede overt anatomical alterations [4].

Visual acuity, although widely used in both clinical practice and research, primarily reflects high-contrast visual performance and does not adequately represent the complexities of everyday vision [7,8,9,10]. Contrast sensitivity (CS), defined as the ability to detect subtle differences in luminance between an object and its background, provides a more comprehensive assessment of visual function [9,10,11]. Activities such as night driving, face recognition, and navigation in low-contrast environments depend heavily on intact CS, making it a critical yet often overlooked component of visual performance [12,13,14].

New evidence suggests that functional impairments in CS may occur in diabetic patients even in the absence of clinically detectable DR [15,16]. This has caused a paradigm shift in the understanding of DR, from a solely microvascular disorder to a complicated neurovascular disease involving early retinal neuronal dysfunction [17,18]. Experimental and clinical studies have demonstrated that diabetes-induced metabolic stress, oxidative damage, and low-grade inflammation contribute to retinal neurodegeneration, affecting ganglion cells and inner retinal layers [19,20,21]. These early alterations may disrupt visual processing pathways [22,23], resulting in measurable deficits in CS before the appearance of classical fundoscopic signs [15].

In addition to its potential role as an early biomarker, CS has been shown to correlate with the severity of DR and the presence of diabetic macular edema (DME) [24,25,26], a major cause of vision loss in diabetic patients [27]. Evidence further indicates that CS deficits may already be present in individuals with diabetes who do not exhibit clinically detectable signs of retinopathy, with reductions observed across multiple spatial frequencies, including intermediate and higher ranges [28]. Moreover, CS testing has been shown to effectively distinguish diabetic individuals from healthy controls, although its ability to differentiate between various stages of retinopathy appears to be more limited [29].

Despite growing interest, CS testing is not routinely incorporated into standard ophthalmologic evaluation for diabetic patients. Limitations related to test standardization, variability, and accessibility have hindered its widespread adoption [30]. Nonetheless, innovations in digital testing platforms and imaging technology provide new opportunities to incorporate CS into clinical practices and research protocols [31,32,33].

The aim of this narrative review is to synthesize current evidence regarding the role of CS in diabetic retinopathy, with a focus on its utility as an early functional biomarker, its relationship with retinal structural changes, and its potential applications in clinical practice. This study intends to highlight the value of CS as a supplementary tool in the comprehensive assessment of diabetic retinal disease by bridging the gap between structural changes and functional visual outcomes.

## 2. Materials and Methods

The literature search was performed in PubMed, Scopus, and Google Scholar, covering publications from January 2000 to May 2026, by two authors independently. The search terms included combinations of the following keywords and their Boolean variants: “contrast sensitivity”, “diabetic retinopathy”, “diabetes mellitus”, “visual function”, “retinal neurodegeneration”, “optical coherence tomography”, “OCT angiography”, “diabetic macular edema”, “visual acuity”, and “retinal biomarkers”. Reference lists of the retrieved articles and relevant reviews were also manually screened to identify additional eligible studies.

Original research articles and systematic reviews published in English were considered for inclusion if they addressed contrast sensitivity or contrast sensitivity function in the context of diabetes mellitus or diabetic retinopathy. Studies were excluded if they were case reports, conference abstracts without full text, or if they addressed contrast sensitivity exclusively in ocular conditions unrelated to diabetes.

Titles and abstracts were screened for relevance, and full texts of potentially eligible articles were subsequently reviewed by the authors. Data extraction was performed manually by two other authors, who independently retrieved sample size, study design, diabetic retinopathy stage, CS testing modality, other testing methods (if used), lighting/testing conditions, main quantitative findings, and study limitations from each included article; discrepancies were resolved by discussion.

## 3. Pathophysiology of Visual Dysfunction in Diabetic Retinopathy

The pathophysiology of visual dysfunction in DR involves both microvascular impairment and retinal neurodegeneration, with neuronal dysfunction now recognized to occur early, often before clinically detectable vascular abnormalities. This neurovascular view of DR is directly relevant to CS, since contrast processing depends on both the vascular supply to the retina and the integrity of the neural elements that encode and transmit luminance differences.

### 3.1. Hyperglycemia-Induced Metabolic Pathways

Chronic hyperglycemia induces a variety of metabolic processes that contribute to the development of DR, such as the polyol pathway, advanced glycation end-product (AGE) production, protein kinase C (PKC) activation, and the hexosamine pathway, together causing oxidative stress, inflammation, and cellular dysfunction in the retina [34].

In the polyol pathway, excess glucose is converted to sorbitol via aldose reductase, reducing antioxidant capacity and increasing oxidative damage [35]; inhibition of aldose reductase attenuates retinal injury [36,37,38], and increased enzyme activity is linked to cellular degeneration under hyperglycemic conditions [39,40].

AGEs accumulate in retinal tissues, causing structural and functional changes through protein crosslinking, oxidative stress, and pro-inflammatory signaling [41], and contribute to extracellular matrix remodeling that impairs vascular integrity [42,43].

PKC activation, especially of the PKC-β isoform, promotes endothelial dysfunction, increased vascular permeability, leukostasis, inflammation, and driven vascular leakage [44,45,46], while increased hexosamine pathway flux causes abnormal protein glycosylation and altered gene expression, contributing to oxidative stress, inflammation, and apoptosis [44,47]. These converging metabolic disturbances contribute to the retinal cellular dysfunction that underlies later CS impairment.

### 3.2. Retinal Neurodegeneration

Retinal neurodegeneration is an early and significant component of DR, typically preceding clinically evident vascular changes [48], and it is characterized by progressive ganglion cell damage, amacrine/bipolar cell dysfunction, thinning of the inner retinal layers, including the retinal nerve fiber layer (RNFL), and neuronal apoptosis [20,49,50], driven by chronic hyperglycemia-related oxidative stress, mitochondrial dysfunction, inadequate neurotrophic support, and low-grade inflammation of the microglia [50,51]. As CS depends on the integrity of both retinal and post-retinal neural processing, early neuronal impairment may result in measurable functional deficits even in the absence of overt microvascular damage [15,52].

### 3.3. Microvascular Alterations

Early in DR, reduced retinal perfusion from vasoconstriction of retinal arteries and arterioles impairs blood flow and metabolic imbalance [53,54] contributing to endothelial dysfunction, increased vascular permeability, and disruption of the blood–retinal barrier [54,55]. These are further exacerbated by ion channel-related alterations [56,57].

Pericyte loss, a key feature of diabetic microangiopathy, results in capillary instability, endothelial degeneration, and altered perfusion [53,57,58,59], while chronic inflammation promotes leukostasis and capillary occlusion leading to progressive retinal ischemia and hypoxia [57,60,61,62].

Hypoxia-driven VEGF production promotes vascular leakage and retinal edema [63], while structural remodeling of the capillary basement membrane further disrupts vascular integrity [57,64,65]. By reducing perfusion and oxygen delivery to the inner retina, the microvascular changes compromise the metabolic support required for normal neural contrast processing, providing a vascular contribution to CS impairment alongside the neurodegenerative mechanisms.

**Figure 1:**
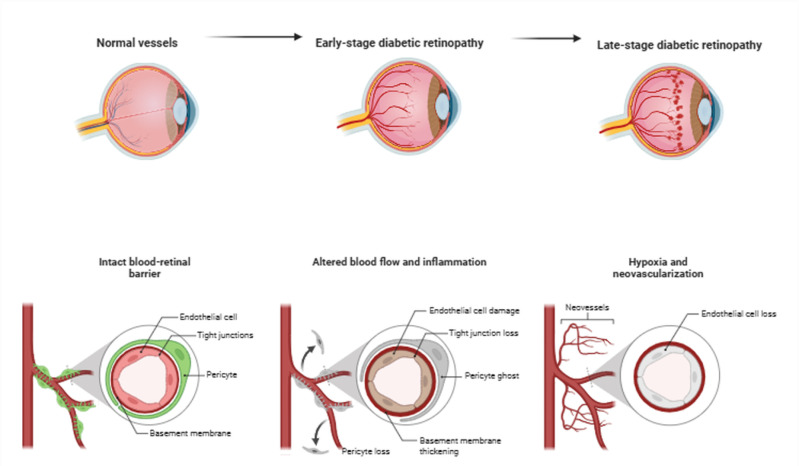
Pathological vascular changes in DR. Created with BioRender.com.

These changes collectively cause increasing microvascular instability, impaired perfusion, and ischemia, all of which contribute to retinal impairment (Figure 1).

## 4. Contrast Sensitivity: Principles and Methods of Assessment

Contrast sensitivity represents a key component of visual function, defined as the ability to detect differences in luminance between an object and its background [66]. Unlike visual acuity, which primarily assesses the resolution of high-contrast stimuli, CS reflects the performance of the visual system under more realistic conditions, where contrast is often reduced [66,67]. This makes it particularly relevant in the evaluation of functional visual impairment in patients with DR, in whom early deficits may not be captured by standard acuity testing [15].

Contrast sensitivity is not a single fixed value but varies depending on the spatial frequency of the visual stimulus [67]. Spatial frequency refers to the level of detail present in an image, with low spatial frequencies corresponding to large objects and general shapes, and high spatial frequencies corresponding to fine details and edges. The relationship between CS and spatial frequency is described by the contrast sensitivity function (CSF), which typically exhibits an inverted U-shaped profile [68,69]. Sensitivity is highest at intermediate spatial frequencies and decreases toward both lower and higher extremes [70,71]. In diabetic patients, alterations in the CSF may occur selectively at specific spatial frequencies, reflecting differential vulnerability of visual pathways [28]. Early neurodegenerative changes, particularly involving inner retinal neurons, may preferentially impair the processing of low-contrast and intermediate-frequency stimuli, leading to subtle but functionally significant visual deficits [10].

Several methods have been developed to assess CS in clinical and research settings, each with distinct characteristics. The Pelli–Robson chart is one of the most widely used tools in clinical practice [72]. It evaluates CS at a single, relatively low spatial frequency using letters of constant size that progressively decrease in contrast [72,73]. Its simplicity, rapid administration, and good reproducibility make it suitable for routine use; however, its limitation lies in the inability to capture the full contrast sensitivity function, which may reduce its sensitivity to subtle or frequency-specific deficits [74,75].

More comprehensive assessments can be achieved using instruments such as the CSV-1000 or the Functional Acuity Contrast Test (FACT), which evaluate CS across multiple spatial frequencies using sine-wave gratings. These methods provide a more detailed characterization of the CSF and are therefore particularly valuable in research settings. However, their use in routine clinical practice is limited by longer testing times and the need for specialized equipment [67,76,77].

An alternative approach involves low-contrast visual acuity (LCVA) testing, which utilizes standard optotype charts, such as ETDRS charts, presented at reduced contrast levels [8,78]. This method is easy to implement and integrates well into existing clinical workflows, offering a practical compromise between simplicity and functional relevance. Nevertheless, LCVA measurements remain influenced by visual acuity and do not fully isolate CS, limiting their precision compared to dedicated CSF-based assessments [78].

More recently, digital and computer-based CS tests have emerged, enabling automated assessment using calibrated screens or portable devices [79,80,81,82]. These technologies offer potential advantages in terms of standardization, automated scoring, and remote monitoring, making them particularly attractive for telemedicine applications [79]. However, variability in device calibration and the limited validation of some platforms remain important challenges that need to be addressed before widespread clinical adoption.

Each approach carries distinct trade-offs between practicality and precision, which in turn determine its suitability for routine clinical use versus research applications. Letter-based charts (Pelli–Robson, low-contrast Sloan optotypes) and sine-wave grating charts (CSV-1000) are inexpensive, quick to administer, and require minimal operator training, making them realistic candidates for incorporation into standard diabetic eye examinations. Computerized adaptive methods, such as the qCSF or Rabin contrast sensitivity test, offer greater precision and a wider dynamic range but depend on dedicated hardware and software, currently limiting their use to research or specialized clinical settings rather than large-scale screening. Table 1 synthesizes the methodological characteristics across the contrast sensitivity tests discussed [8,28,73,77,80,81,82,83]. 

**Table 1:** Contrast sensitivity tests and their characteristics.

Method	Spatial Frequency Coverage	Feasibility	Reproducibility	Clinical Applicability	Limitations
Pelli–Robson chart	Single, low spatial frequency	Quick (2–3 min), minimal training, inexpensive	Good test–retest reliability	Widely used in routine ophthalmologic practice	Cannot capture the full CSF; insensitive to frequency-specific deficits
CSV-1000	4 discrete frequencies (3, 6, 12, 18 cpd)	Backlit chart, fixed distance, few minutes per eye	Discrete contrast steps limit precision; systematic disagreement with other sine-wave grating devices, especially at low spatial frequencies	Feasible in clinic and research; more informative than single-frequency charts	Inter-device variability (even among similar sine-wave instruments) limits cross-study comparability
FACT (Functional Acuity Contrast Test)	5 frequencies (1.5, 3, 6, 12, 18 cpd), sine-wave gratings	Chart-based, similar format to CSV-1000	Better test–retest agreement than its predecessor (Vistech), but repeatability declines at higher spatial frequencies	Mainly research and specialized/refractive surgery settings; no studies using FACT were identified among the diabetic-retinopathy evidence reviewed	Reduced repeatability at higher frequencies; ceiling effects reported in some populations
Low-contrast visual acuity (LCVA, Sloan charts)	Not frequency-specific (letter-acuity based, at fixed reduced contrast levels)	Integrates directly into existing ETDRS-style workflows	Good reproducibility	Practical, easy to add to standard acuity testing	Remains influenced by visual acuity; does not isolate CS as a distinct functional measure
Computerized Gabor patch testing (photopic/mesopic)	Wide, continuous range; can test under multiple luminance conditions	Requires calibrated monitor, controlled room lighting, longer session (dark adaptation)	Reliable within-study, but luminance and testing protocols vary substantially between studies	Mainly research; valuable for detecting mesopic-specific deficits	Equipment-dependent; luminance not standardized across studies
Quantitative CSF (qCSF)	Full CSF estimated across a wide frequency and contrast range	Specialized software/hardware (Sentio platform, tablet-based), trained operator	High precision; strong test–retest reliability	Currently limited to research or specialized clinics; not yet practical for large-scale screening	Cost and equipment access are the main barriers to routine adoption

Overall, while there are several ways to measure CS, the lack of standardization among testing modalities is a serious disadvantage. This inconsistency makes comparisons across research difficult and impedes the incorporation of contrast sensitivity into standard clinical practice.

## 5. Contrast Sensitivity Impairment in Diabetic Patients

A large body of research shows that CS is impaired in DR patients at different stages of the disease. Importantly, functional losses frequently precede measured reductions in visual acuity.

**Figure 2:**
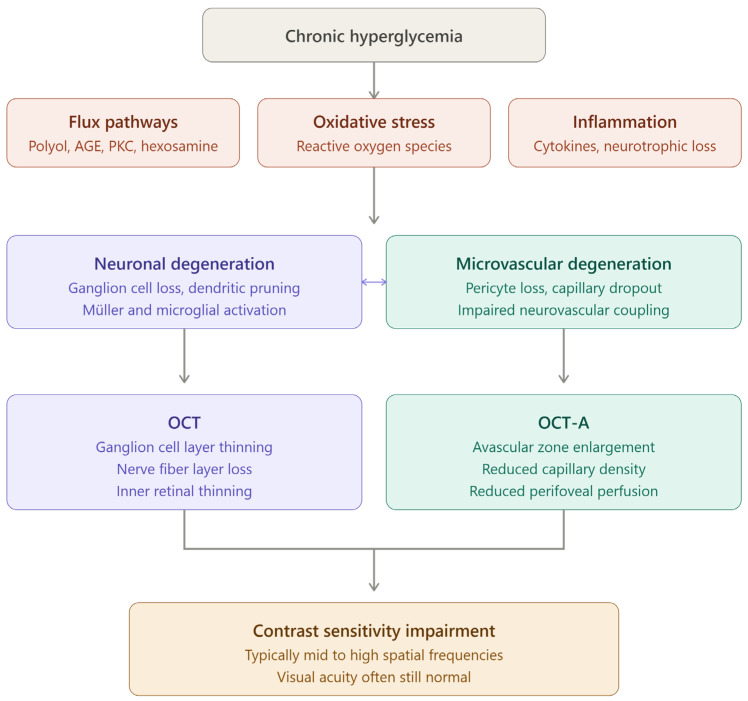
Conceptual figure linking diabetic metabolic injury, retinal neurodegeneration, OCT and OCT-A changes, and contrast sensitivity impairment.

Population-based research has shown that CS impairment in diabetes is driven not only by retinal structural changes, but also by the severity of systemic and ocular diseases. Reduced CS has been associated with longer diabetes duration, poor glycemic control, diabetic neuropathy, anemia, cataract severity, and increasing stages of diabetic retinopathy. In multivariate analyses, visual acuity, neuropathy severity, cataract grade, diabetic retinopathy stage, and age were identified as independent predictors of reduced contrast sensitivity. CS deterioration was also observed even in patients without advanced retinal thickening [84,85,86]. Research supports a unifying framework in which hyperglycemia-driven metabolic injury acts on the retinal neurovascular unit to produce parallel neuronal and microvascular degeneration, which is detectable structurally on OCT and OCT-A and expressed functionally as CS loss well before visual acuity declines (Figure 2).

### 5.1. Contrast Sensitivity in Diabetic Patients Without Clinically Visible Retinopathy

Several studies have demonstrated that CS may be reduced in individuals with diabetes even in the absence of clinically detectable retinopathy [87]. Subtle CS abnormalities have also been reported in individuals with prediabetes [88]. CS testing performed under both moderate and dim illumination conditions has been proposed as a useful approach for detecting subtle visual dysfunction, as reduced luminance may increase the sensitivity of the test to early retinal abnormalities [28]. The observed deficits are often subtle and may affect specific spatial frequencies. In this context, CS has been shown to be reduced despite normal visual acuity, particularly at increasing testing distances, and to correlate negatively with both HbA1c levels and duration of diabetes, indicating an association between metabolic control and early functional impairment [89]. Similarly, studies in children with type 1 diabetes without visible retinopathy demonstrated abnormalities in chromatic CS [90], together with structural retinal alterations on optical coherence tomography (OCT) [91]. Additional evidence suggests that these early abnormalities may become more apparent under mesopic conditions. Studies using computerized CS testing with Gabor patches demonstrated significantly reduced mesopic foveal CS in type 2 diabetic patients without clinically visible retinopathy, despite preserved visual acuity, normal fundus examination, and normal OCT findings [82]. Psychophysical studies have also demonstrated reduced CS in diabetic patients with no DR [92], with evidence suggesting that increased internal visual noise may contribute to these deficits and may correlate with disease duration and reduced retinal sensitivity [93]. However, other studies found no significant differences between healthy individuals and diabetic patients without retinopathy [94].

This early impairment is thought to be primarily driven by retinal neurodegeneration. Data indicate that hyperglycemia-induced oxidative stress, mitochondrial dysfunction, and low-grade inflammation contribute to damage of retinal ganglion cells and inner retinal layers [95]. As these structures play a critical role in contrast detection, their dysfunction may lead to measurable reductions in CS even when vascular changes are not yet apparent.

### 5.2. Contrast Sensitivity in Non-Proliferative Diabetic Retinopathy

As DR progresses to the non-proliferative stage, CS impairment becomes more pronounced and more consistently detectable. The degree of CS reduction has been shown to correlate with the severity of retinopathy, suggesting a progressive decline in visual function alongside increasing structural damage. In patients with moderate-to-severe diabetic retinopathy and low vision, CS showed a significant inverse correlation with LogMAR visual acuity [96].

In a cross-sectional study which included diabetic patients without retinopathy, mild NPDR, and healthy controls, CS was significantly reduced in diabetic individuals, with greater impairment in mild NPDR. Importantly, CS was already decreased in patients without clinically detectable retinopathy, despite preserved visual acuity, indicating early functional impairment [26]. In another study, quantitative CS function testing showed a progressive reduction in CS across diabetic retinal disease stages. The decline was evident not only in global metrics, such as the area under the logarithmic contrast sensitivity function (AULCSF) and contrast acuity, but also across several spatial frequencies, particularly at higher frequencies [25]. Contrast sensitivity and low-contrast visual acuity have been found significantly impaired in patients with non-proliferative diabetic retinopathy, whereas high-contrast visual acuity did not differ between patients with no DR and healthy individuals [94].

A recent systematic review further supported the clinical relevance of CS assessment in diabetes, showing that most studies reported significant reductions in CS in patients with diabetes and DR compared with healthy controls, regardless of the testing method used [16].

### 5.3. Contrast Sensitivity in Diabetic Macular Edema

In patients with DME, CS impairment may be more severe. The accumulation of intraretinal fluid disrupts photoreceptor alignment, alters synaptic transmission, and degrades the quality of visual signals reaching higher visual pathways [27,97]. As a result, patients with DME often exhibit marked reductions in CS, which may significantly impact visual function and quality of life. Furthermore, recent OCT-angiography (OCT-A)-based studies have demonstrated strong associations between CS impairment and microvascular alterations in DME, with CS showing larger effect sizes and stronger structure–function correlations than visual acuity [98].

Studies evaluating anti-VEGF therapy in patients with diabetic macular edema have shown that CS improves following treatment and may be more sensitive than best-corrected visual acuity in detecting functional changes. In patients with persistent DME switched from ranibizumab to aflibercept, CS measured with the Pelli–Robson chart improved significantly alongside improvements in BCVA and central retinal thickness, while changes in CS demonstrated a stronger correlation with reductions in retinal thickness than changes in visual acuity. Similarly, the Mars Letter Contrast Sensitivity (MLCS) test demonstrated greater sensitivity for identifying visual improvement after intravitreal therapy compared with visual acuity and computerized CS testing methods [99,100].

Table 2 summarizes key studies, describing contrast sensitivity methods, patient populations, testing conditions, and main findings reported across the literature on diabetic patients without clinically visible retinopathy, with or without non-proliferative disease.

**Table 2:** Representative studies evaluating contrast sensitivity impairment in diabetes mellitus.

Authors, Year	Study Type	Population (Sample Size)	CS Method	Testing Conditions	Imaging Method	Main Findings	Limitations
Georgakopoulos et al., 2011 [90]	Case–control	60 children and adolescents with type 1 diabetes without DR versus 45 age- and gender-matched healthy controls	CSV-1000 contrast sensitivity testing at multiple spatial frequencies	photopic, monocular, 2.5 m	None	Significant reduction in CS at all spatial frequencies despite absence of clinically visible diabetic retinopathy; HbA1c inversely correlated with CS	No structural/imaging correlation performed
Katz et al., 2010 [82]	Case–control	9 type 2 diabetic patients (17 eyes) without DR, and 7 healthy controls (14 eyes)	Computerized Gabor-target 4-AFC test (staircase method), SF 3–12 cpd	Photopic (20 cd/m^2^, dark room) and mesopic (0.9 cd/m^2^, neutral density filter); monocular, natural pupils, viewing distance 150 cm	OCT (Stratus, Carl Zeiss Meditec)—foveal avascular zone height and adjacent retinal maximal height	Mesopic CS was significantly reduced in diabetic patients despite normal OCT, normal fundus findings, and preserved visual acuity	Small, relatively homogeneous sample; no correlation found with HbA1c or duration, possibly due to sample size
Joltikov et al., 2018 [81]	Case–control	57 diabetic patients without DR, mild NPDR, and moderate NPDR, and 18 healthy controls	Quick Contrast Sensitivity, AST Sentio Platform; AULCSF integrated 1.5–18 cpd	Monocular, tested after BCVA measurement, untested eye patched	SD-OCT (Spectralis HRA+OCT)	CS abnormalities detected earlier than visual acuity changes; OCT showed ganglion cell and inner plexiform layer thinning	Modest sample size; sex distribution imbalance across groups; lower-density SD-OCT scans may have limited DRIL detection sensitivity; larger 6 mm measurement ring limits comparability with prior studies (1–3 mm rings).
Lupión Durán et al., 2021 [94]	Cross-sectional	196 diabetic patients without DR, 114 diabetic patients with NPDR, and 58 healthy controls	Pelli–Robson contrast sensitivity test and low-contrast visual acuity charts (1.25%, 2.5%, and 5%)	Pelli–Robson at 1 m, illumination 90–120 cd/m^2^; Sloan test at 2 m; ETDRS high-contrast VA at 4 m	none	Patients with NPDR demonstrated significantly worse contrast sensitivity and low-contrast visual acuity despite preserved high-contrast visual acuity	Sample size still limited for subgroup analysis; ambient lighting/brightness difficult to fully standardize across exams
Pramanik et al., 2020 [15]	Cross-sectional	30 diabetic patients without DR, 43 diabetic patients with mild NPDR, and 35 healthy controls	Rabin contrast sensitivity test (log CS, 8 contrast levels, 0.25 log CS steps/row)	Illuminator cabinet, luminance 170 cd/m^2^; monocular, best correction, dark room, viewing distance 4 m; worse eye value used	SD-OCT (Heidelberg Spectralis)	Significant reduction in CS in diabetic groups despite no significant differences in central macular thickness or visual acuity	Relatively small sample size per subgroup; only VA and CS assessed (no color vision, dark adaptation, or other psychophysical tests)
Safi et al., 2017 [28]	Cross-sectional	46 type 2/2 type 1 diabetic patients without DR and 46 healthy controls	CSV-1000 backlit chart, 4 spatial frequencies (3, 6, 12, and 18 cpd)	Monocular, BCVA correction, 2.5 m; two lighting conditions: moderate and dim (25 min dark adaptation); luminance 85 cd/m^2^	None	Diabetic subjects showed significant CS reduction at nearly all spatial frequencies under both lighting conditions, despite absence of retinopathy; combined photopic and mesopic testing achieved good discriminative accuracy	Limited number of type 1 diabetes subjects prevented analysis by diabetes type; no OCT performed to exclude subclinical DME; MPOD not measured, limiting mechanistic interpretation

## 6. Structure–Function Relationship

A thorough assessment of DR requires an understanding of the connection between structural retinal changes and functional vision impairment. In-depth, layer-specific evaluation of the retina’s neuronal and vascular components has been made possible by advancements in retinal imaging, offering important insight into the mechanisms underlying CS impairment [4].

OCT has been used in revealing early neurodegenerative changes in diabetic patients. Several studies have demonstrated thinning of the retinal nerve fiber layer (RNFL) and ganglion cell layer (GCL), even in individuals without clinically apparent retinopathy [101,102]. These inner retinal layers play a critical role in visual signal transmission and contrast processing, and their structural compromise has been consistently associated with reductions in CS [101]. Ganglion cell loss may disrupt spatial contrast encoding, contributing to deficits that are not detected by standard visual acuity testing [103]. The correlation between inner retinal thinning and CS impairment supports the hypothesis that neuronal damage is a key driver of early functional loss in diabetes. Structural disorganization of the inner retina has also emerged as an important imaging biomarker associated with functional impairment in diabetes [104]. Large cohort data further support early neuroretinal involvement in diabetes without clinically visible retinopathy, showing reduced Pelli–Robson CS together with thinning of the ganglion cell layer and inner plexiform layer [105]. Disorganization of the retinal inner layers (DRIL), identified on OCT as loss of clear boundaries between inner retinal layers, has been associated with reduced contrast sensitivity function, decreased visual acuity, and impaired visual field performance in diabetic patients without advanced diabetic retinopathy or DME. Moreover, DRIL has been detected even in some diabetic patients without clinically visible retinopathy, supporting the concept that neuroretinal dysfunction and structural neuronal alterations may precede overt microvascular disease [81].

In addition to neuronal alterations, microvascular changes assessed by OCT-A have shown significant associations with visual function. OCT-A enables non-invasive visualization of the retinal capillary plexuses, allowing quantification of vessel density and perfusion parameters. Capillary dropout and impaired perfusion lead to localized retinal hypoxia, which further exacerbates neuronal dysfunction and contributes to the deterioration of visual processing [22]. Reduced superficial capillary plexus vessel density (SCP VD) has been associated with worsening diabetic retinopathy severity and decreased CS, particularly in the nasal and temporal retinal quadrants. An enlarged foveal avascular zone (FAZ) area was also correlated with functional deterioration and increasing disease severity. Structural OCT analysis demonstrated thinning of the RNFL and GCL, especially in early diabetic retinal disease, suggesting progressive neurodegenerative involvement. Furthermore, positive correlations between SCP vessel density and ganglion cell layer thickness support the concept of close neurovascular coupling in diabetic retinal dysfunction [106].

Studies have shown that AULCSF is significantly reduced even in diabetic patients without retinopathy and in early NPDR, while visual acuity remains largely preserved. Moreover, CS exhibits stronger correlations with microvascular parameters, such as vessel density and capillary flow indices compared to visual acuity, and performs better in discriminating between disease stages [107]. Ha et al. showed that the capillary flux index of the radial peripapillary capillary plexus was significantly associated with multiple contrast sensitivity parameters, including AULCSF, contrast acuity, and CS at 6, 12, and 18 cycles/degree, whereas visual acuity showed a weaker and more limited association. In contrast, capillary perfusion density was not significantly associated with CS outcomes [25]. Importantly, the relationship between structural and functional changes is not always linear. In some cases, significant structural abnormalities may be present with relatively preserved functional measures, while in others, functional deficits such as reduced CS may occur in the absence of overt structural damage detectable by current imaging techniques [15,108]. Studies demonstrated that, while OCT-derived values were similar, CS progressively decreased from diabetic subjects without retinopathy to those with mild NPDR [15]. This discrepancy may reflect the limited resolution of imaging modalities, early synaptic dysfunction, or alterations in post-retinal visual pathways. In the study of Ostadimoghadam et al. no significant correlation was found between CS and OCT-derived retinal thickness parameters [26].

## 7. Discussion

The available evidence suggests that CS impairment represents an early and clinically relevant manifestation of diabetic retinal dysfunction, often preceding measurable reductions in visual acuity or clinically visible retinopathy. CS showed discriminatory ability between healthy controls and diabetics without retinopathy, and to a lesser extent between diabetics without retinopathy and mild NPDR, supporting its value as an early functional marker [15].

Despite its clinical relevance, CS testing presents several limitations that currently restrict its widespread integration into routine ophthalmologic practice for patients with DR. These limitations relate to methodological variability, susceptibility to confounding factors, and the lack of standardized testing protocols [109,110].

One of the primary challenges lies in the heterogeneity of available testing methods. Different instruments assess CS under varying conditions and across different spatial frequencies, making direct comparison between studies difficult [109]. Chart-based methods such as the Pelli–Robson test evaluate CS at a single spatial frequency [111], whereas CSV-1000 provides multifrequency assessment [112]. This variability of the reviewed studies affects data interpretation and restricts the capacity to establish widely accepted reference values. Beyond instrument type, testing conditions themselves varied substantially across studies. Luminance and testing distance were rarely matched: CSV-1000 was typically administered at a fixed distance under approximately 85 cd/m^2^, or alternatively under separate moderate and dim lighting conditions; the Pelli–Robson test was performed at 90–120 cd/m^2^; and computerized Gabor-based testing spanned a considerably wider range, from 20 cd/m^2^ under photopic conditions to 0.9 cd/m^2^ under mesopic conditions. This lack of consistency further limits comparability between studies and complicates translation into a single clinical protocol. A minimum standardization would therefore include a fixed testing distance, calibrated luminance for both photopic and mesopic conditions since mesopic CS appears to be preferentially affected early in some studies, monocular testing with the eye’s best correction, and a fixed adaptation period before mesopic or dim testing, commonly reported as 15–25 min across studies.

The heterogeneity becomes clearer when the evidence is examined by test category rather than pooled together. In the largest cohort using the Pelli–Robson chart, CS was significantly reduced only in eyes with established NDPR compared with both controls and diabetic patients without retinopathy, with no difference between the latter two groups [94], suggesting that single-frequency chart testing may be less sensitive to pre-clinical neuroretinal dysfunction and only becomes abnormal once retinopathy is clinically detectable. Low-contrast visual acuity testing with Sloan charts in the same cohort followed the same pattern [94]. CSV-1000 multifrequency testing detected impairment even in diabetic patients without any visible retinopathy, with reductions present at all tested spatial frequencies in one cohort and inversely correlated with glycosylated hemoglobin [90], while another cohort found reductions at all frequencies except the lowest under moderate lighting, with no correlation to HbA1c [28]. Computerized adaptive CSF testing went further still, detecting significantly reduced area under the log CSF in diabetic subjects with DRIL compared with both controls and diabetics without DRIL, even before proliferative disease or macular edema was present [81]. Mesopic and dim-light testing highlighted early signals in diabetic patients without retinopathy, where one cohort found reduced CS under mesopic but not photopic conditions at low spatial frequency [82], while another found reductions across a broader spatial frequency range specifically under dim-light conditions, with a magnitude of loss comparable to that seen under standard illumination [28]. Taken together, this breakdown suggests that the apparent inconsistency in the literature does not necessarily reflect contradictory findings, but rather that different instruments carry different sensitivity thresholds, with mesopic and adaptive methods appearing more capable of detecting pre-clinical dysfunction, while single-frequency photopic chart tests may only become abnormal once retinopathy is already clinically visible.

CS measurements are influenced by several patient-related and environmental factors. Age-related changes in visual function are known to reduce CS, even in healthy individuals, which may confound the assessment in diabetic populations [113,114]. Optical factors, such as uncorrected refractive errors and media opacities, can significantly affect test performance by reducing retinal image quality. To address these confounders, most protocols exclude eyes with clinically significant cataract or refractive error beyond a defined threshold; while methodologically necessary, this approach limits generalizability to the diabetic population that CS testing is ultimately intended to serve, where early lens changes are common. Furthermore, testing conditions such as ambient illumination, screen calibration in digital systems, and patient cooperation can introduce variability and reduce reproducibility [114,115]. Unlike visual acuity, which benefits from well-established guidelines and widespread familiarity, CS assessment lacks uniformity. As a result, its clinical utility may vary between institutions, and its integration into routine workflows remains inconsistent [30]. Beyond glycemic control itself, the choice of antidiabetic medication may also influence ocular outcomes in patients with diabetes [116]. This is relevant to the overall management of diabetic eye disease and represents an additional source of variability that was not accounted for in the CS studies. Across studies, reported reductions are frequently small in absolute terms and, while statistically significant at the group level, have in some cases been explicitly described as not clinically significant or insufficient, on their own, to reliably discriminate disease onset in an individual patient. This distinction between statistical and clinical significance is a critical gap: without an established minimal clinically important difference, CS reductions remain informative for research comparisons but are not yet actionable as a standalone clinical threshold for diagnosis or monitoring.

Finally, while CS provides valuable information about visual function, it does not fully capture all aspects of visual performance, and its relationship to structural imaging is not always straightforward; in several studies, CS was significantly reduced even when central macular thickness and visual acuity showed no differences between groups, suggesting it may capture functional deficits not reflected in conventional OCT-based structural measures. It should therefore be considered as a complementary tool rather than a replacement for other functional and structural assessments [67,80].

## 8. Future Directions

The growing recognition of CS as a potential marker of functional visual impairment in DR shows the necessity for its integration into research and therapeutic practice. One of the most promising possibilities is the combination of CS with retinal imaging techniques. Multimodal techniques that include OCT, OCT-A, and functional testing may provide a more complete assessment of the retina. Such integration could improve the identification of early disease stages and allow more accurate monitoring of progression.

Large-scale studies combining multiple visual function measures, with CS, visual acuity, reading performance, low-luminance vision, perimetry, and microperimetry, have demonstrated high accuracy in classifying diabetes and different stages of diabetic retinopathy using ensemble machine learning models [117]. In these approaches, CS contributed as part of a broader functional assessment strategy.

Artificial intelligence is expected to play a role in this context. Machine learning algorithms applied to multimodal datasets may enable the detection of subtle patterns linking structural alterations with functional deficits. These models have the potential to improve risk stratification, forecast disease progression, and provide personalized management methods.

Another area of development is the improvement of digital and portable contrast sensitivity testing. These technologies could allow remote monitoring and incorporation into telemedicine programs, which are especially useful in the management of chronic diseases like diabetes. Expanding availability to functional testing could improve early detection in impoverished communities and lower barriers to routine ophthalmologic evaluation.

Longitudinal studies are needed to further understand the temporal relationship between CS impairment and structural retinal changes. Furthermore, future research should aim to generate normative databases and standardized testing protocols for contrast sensitivity across different populations and clinical settings, making it easier to compare studies and develop clinically meaningful thresholds for diagnosis and monitoring.

## 9. Conclusions

CS shows evidence of impairment in diabetic patients before visual acuity loss or clinically visible retinal lesions are detectable, suggesting it may reflect early retinal dysfunction in diabetic retinopathy. However, the magnitude of this impairment varies considerably across studies depending on the CS method, spatial frequency, and lighting conditions used, and no validated clinically meaningful threshold has yet been established. Standardized testing protocols and longitudinal studies correlating CS changes with the subsequent development of diabetic retinopathy are needed before its integration into routine clinical practice can be supported.

## Data Availability

No new data were created or analyzed in this study.
